# How to Define Situated and Ever‐Transforming Family Configurations? A New Materialist Approach

**DOI:** 10.1111/jftr.12167

**Published:** 2016-12-01

**Authors:** Cornelia Schadler

**Affiliations:** ^1^ University of Vienna

**Keywords:** Close relationships, early parenting, family diversity, family life, family theory, feminist theory

## Abstract

Within the past few decades, the configuration “family” has included diverse living arrangements, yet traditional definitions of family persist. Accordingly, family studies scholars have discussed research strategies and theoretical approaches to define the shifting boundaries of family. In this article I propose the approach of new materialism for a contemporary definition of family that focuses on situated processes and the complex interplay of material‐discursive differentiation processes. This perspective enriches current debates on defining family by adding concepts of intracontextual posthuman practices and multilocal forms of agency to the discussion, thus allowing for a definition of family that helps make comprehensible today's ever‐transforming configurations.

In recent decades, the concept of family has been subjected to redefinition as family scholars have debated the question of who and what constitutes a family. Researchers have suggested, for instance, focusing on family relations (Smart, 2007), meaningful practices (Morgan, 2011; Widmer & Jallinoja, 2008), caring practices (Roseneil & Budgeon, 2004), intentions (Muraco, 2006), kinship systems (Johnson, 2000), togetherness (Stoilova, Roseneil, Crowhurst, Hellesund, & Santos, 2014), intimacy (Weingarten, 1991), personal and situated communities (Jamieson & Simpson, 2013; Pahl & Spencer, 2004), or intersectional power structures (Few‐Demo, 2014). Each of these approaches discusses processes that define the boundaries of relationships or families. Despite many existing forms of close relationship, however, we are experiencing what Braidotti (2002) calls a “schizoid double pull”: Although operational definitions of family have been broadened, traditional concepts of family have not disappeared.

In this article, I discuss a recent nexus of theories, called new materialisms, that focus on multilocal definitions of family, agency, and situated posthuman processes. *New materialism* is a recent term (Dolphijn & van der Tuin, 2012) that is subsuming a number of materialist theories, such as the posthumanism of Donna Haraway (2008), the Deleuzian materialism of Rosi Braidotti (2002, 2006, 2013), and the agential realism of Karen Barad (2007). These concepts currently are widely discussed in science and technology studies and gender studies, but they have not yet caught the attention of most family studies scholars (Alaimo & Hekman, 2007; Coole & Frost, 2010; Schadler, 2014). However, I consider these theories a fruitful contribution to the ongoing discussion about family definitions. Taken together, the approaches represent a paradigm shift away from constructivism and toward materialism (Coole, 2013). New materialist theories assume that (a) family relationships are not the product of context or the product of humans; (b) family configurations are in constant transformation but simultaneously form fixed boundaries; and (c) the material environments and discursive contexts of family processes become a part of the definition of family.

In this article, I first selectively review family research to inform contemporary concepts of family. I then describe the assumptions of new materialism (anti‐dualism, radical immanence, transversality, and posthuman agency) and provide two brief empirical examples from my own research to illustrate the advantages of a new materialist perspective. One example concerns the formation of the nuclear family during the transition to parenthood, and the other a polyamorous relationship within a society that is centered on two‐adult relationships. Finally, I conclude with a discussion of the contributions to family theory and research of a new materialist approach.

## Defining the Boundaries of Families

Although there is no universal notion of what constitutes a family, to do research, family studies scholars explicitly or implicitly rely on a definition to draw the boundary between family and nonfamily. Some scholars use structural processes, such as legal definitions or political definitions, to define who or what a family is; others focus on individual processes, such as meaningful interactions or practices.

Half a century ago Talcott Parsons and Robert Bales (1956) defined the nuclear family as containing a male adult, a female adult, and at least one child to be the social norm. However, since they formulated their model of the nuclear family, many restructurations of North American and European societies have taken place (Beck‐Gernsheim, 1998; Lesthaeghe, 2010; Liefbroer, 2009). On a structural level the so‐called trinity of marriage, sex, and childbirth (Allan, 2008; Beck‐Gernsheim, 1998; Cherlin, 2004) has become disconnected. Women's movements restructured societies and offered women access to the labor market, leading to a decreasing dependence on marriage or partnership (Blossfeld & Drobnic, 2009). Access to birth control, education, living space, and the decrease in norms and institutions that forced women and men into traditional biographies created more space to negotiate life phases and relationships. Divorce has become an acceptable option and has provided escape from unhappy, or even abusive, relationships (Amato, 2010). In North American and Northern European countries, living as a single‐parent family or stepfamily is becoming “normal” and unstigmatized (Williams, 2004). In contrast, family forms that include more than two adults or nonbiological or nonadopted children are rarely represented by legal structures and policies (Klesse, 2014; Roseneil & Budgeon, 2004).

Nevertheless, the lived realities of families and everyday family life are complex (Pahl & Spencer, 2010). For that reason, some scholars have investigated self‐definitions of family boundaries (Widmer & Jallinoja, 2008). Research based on interpretative, ethnomethodological, social constructionist, and symbolic interactionist theories has focused on how individual people and communities narrate and define family. These theories assume that human entities (individuals) have an inherent ability to interact with one another, to constantly process knowledge, and to transform that knowledge into meaning (LaRossa & Reitzes, 2004). Those sense‐making processes (Schütz, 1974), definitions of situations (Thomas, 1937), and ascriptions of meaning (Blumer, 1969) are the foundations for human actions. Therefore, the goal is to use such subjective accounts as a basis for a social definition of family rather than structural, demographic, or institutional characteristics.

Research from this perspective focuses on meaningful practices (Finch, 2007; Jurczyk, 2013; Morgan, 2011) and assumes that family members actively constitute family when they perceive certain actions to be “family” actions even when structural definitions favor other configurations. For instance, Roseneil and colleagues (Crowhurst, Roseneil, Hellesund, Santos, & Stoilova, 2013; Roseneil, 2006; Roseneil & Budgeon, 2004) have investigated intimate practices, care, and support between individuals who, by standard definition, are not family. Examples are best friends who are raising children together, friends and neighbors who give support in times of sickness, and shared households with ex‐lovers and ex‐partners.

Family members are embedded in a network of interdependencies with other family members and are simultaneously able to negotiate their position, their identity, and their relations within these networks. In family systems theory, for example, transactional sequences of behavior and interpretation define the boundaries of family (Whitchurch & Constantine, 1993). As such, empirical research often focuses on the determinants of unstable systems (Johnson, 2000; O'Brien, 2005) or ambiguous boundaries (Boss, 1980; 2007).

From this perspective, family is the active construction of a network of people, and it is not limited to a household, biological, or legal relationship. Individuals (family members), their biographies, and the structures that create, legalize, and name specific family types are constituted within discourses. For example, Pahl and Spencer (2010) described three discourses that constitute chosen families: (a) narratives that define the family of origin as insufficient in meeting certain needs (support, solidarity); (b) narratives that define family relationships as ties that must be chosen freely, and thus people seek close relationships with people they like, regardless of whether they are genetic kin; and (c) narratives in which certain functions or actions define the concept of family (e.g., raising a child), and thus whoever engages in these activities is family. Further, family communication researchers have focused on discourses that define the boundaries of family. For example, Nelson (2014) asserted that the naming of different family forms in family research, such as voluntary kin or chosen families, reifies boundaries between “real” kin and chosen kin and therefore also creates specific types of human family members.

### *Remaining Questions*

The aforementioned approaches have considered the boundaries of family through (intersecting) structures, systemic differentiation processes, meaningful interactions, dialectical processes of structures and interactions, or discourses. Nevertheless, concerning contemporary family definitions some questions remain unanswered: How do we discuss the shifting boundaries of families? How do families exist outside structural definitions and the meaning‐making processes of humans? And if structure and individual are not perceived as separate analytical categories (e.g., in discourse‐oriented poststructuralist and postmodern approaches), how do we discuss the material boundaries of families?

Another remaining question concerns those beings and objects that belong neither to the realm of structure nor to the realm of the human individual, for instance, animals or technological devices. Those beings and objects are a part of everyday family life, and from an interactionist approach humans can assign them the meaning of family, such as when a woman defines her dog as a part of her family. In this case, the animals or objects are passive recipients of meaning rather than perceived as entities that aid in the construction of family. However, from the perspective of new materialism, it is possible to see those beings and objects as also playing a part in carving out the boundaries of family.

## Defining Families and Posthuman Processes: New Materialism

A nexus of theories subsumed under the notion of new materialism, recently discussed in philosophy of science and gender studies, is increasingly being recognized outside of those disciplines (Coole & Frost, 2010). Theories such as the agential realism of Barad (2003, 2007), the posthumanism of Haraway (1992, 2008), and the Deleuzian materialism of Braidotti (2002, 2006) claim to rewrite definitions of humans, overcome dualisms, include material processes in research, and redefine the relationship of structure and agency in research. These theoretical developments are of interest for contemporary research on what constitutes a family. New materialisms are process ontologies that share the following theoretical foundations: (a) anti‐dualism, (b) radical immanence, (c) transversality, and (d) a posthuman concept of agency.

### *Anti‐Dualism*

Dualisms assume a binary opposition between two separate categories, such as the difference between meaning and action, structure and individual, human and nonhuman, and nature and culture. They assume, for instance, that meaning and action are two areas that interact with each other but are separate realms. Meaning influences actions and actions influence meaning. Nevertheless, both are assumed to be categories that can be observed and analyzed separately.

New materialist researchers attempt to think outside of those dualisms, especially the dualisms of nature and culture and human and nonhuman (Coole, 2013). Thinking in dualisms assumes that nature (the environment, the human body) exists separate from culture (society, meaning). Research practices are then able to represent nature through scientific methods; hence, cultural practices can represent nature. Haraway (1987, 1992, 1993, 1997, 2008) has questioned this process, showing how the dualism of nature and culture is itself created by science. In her work Haraway repeatedly has shown that the dualism of nature and culture was made within techno‐scientific practices. Haraway (1992) argued in her contribution “Promises of Monsters”: “If organisms are natural objects, it is crucial to remember that organisms are not born; they are made in world‐changing technoscientific practices by particular collective actors in particular times and places” (p. 297). She has shown how, for instance, the differences between species are produced within the laboratory as “natural” differences. Nature cannot be understood without the boundary‐making practices of research. Therefore, nature is artificial and hence inseparable from culture as “natureculture.” She has described how humans (and their “natural bodies”) were made and enacted in the scientific practices of taxonomy, demography, and physiology that differentiate humans from other species or other kinds of humans (Haraway, 1993). Hence, humans are not “naturally” different from other species, or different humans are not “naturally” different from each other, but techno‐scientific processes help make these differences. Within those practices nature is not just “constructed” by humans in an epistemic process; it is literally figured materially and semiotically in a space (e.g., the laboratory) where humans, instruments, tubes, mathematic formulas, or computer software act together. Those processes are then excluded from the picture to establish a seemingly natural entity that preexisted its scientific investigation. However, if these techno‐scientific processes become visible, nature and culture become inseparable. This example not only deconstructs the dualism of nature and culture into naturecultures but also redefines the naturecultural entities resulting from these processes as dynamic, material, and corporeal entities.

Theories of social processes often distinguish between a presocial biological body and an immaterial social personality (body–mind dualism). Haraway (1992) reminds us that this is often related to a rhetoric of two distinguishable births: a biological and a social birth (during socialization). Parsons and Bales (1956) are a good example of this way of thinking. They suggest that the “human personality is not ‘born’ but must be ‘made’ through the socialization process and is why families are necessary. They are ‘factories’ which produce human personalities” (Parsons & Bales, 1956, p. 16). Parsons and Bales separate a mere biological reproduction process from a process of socialization after birth. However, everyday reproduction is a highly cultural procedure that includes not just the human bodies but also numerous devices and knowledge, such as cycle charts and pregnancy tests (Schadler, 2014). At the same time, socialization practices start even before pregnancy is confirmed, and they are entangled with the material development of the embryo and fetus. From this perspective the human cannot be separated into a presocial biological body and an immaterial social personality. The human is also defined as a naturecultural entity.

A final important dualism concerns the difference between humans and nonhumans. As described already, new materialist authors keep in mind the boundary‐making practices that are linked to a specific entity. By doing so, the entities become visible as entities that are highly interconnected with their environments. As a consequence, the boundaries between humans and their companions erode (Haraway, 1992). Even if humans seemingly have skin that acts as a corporeal boundary, they are figured by and in steady interconnection with their environment, and they cannot be perceived and described without it. The boundaries between individuals and their (nonhuman) environments become unclear.

### *Radical Immanence*

The issue of radical immanence also concerns the dualism between outside and inside. New materialist authors do not assume preexisting, a priori, or transcendent entities. There is no position outside the process, such as an original substance, or an idea or a god that is causing the process. All positions, entities, and processes are already inside the “phenomenon” (Barad, 2007). Barad (2007) understands the world as a constant material‐discursive differentiation process. A part of this process, for instance, nature, a human, a discourse, or an object has a specific position within that process. This part is connected to all the other entities within the process because their boundaries were all established within this differentiation process. The entities, however, do not link to entities outside the process that cause or alter the position of an entity within the differentiation process. Because all entities are positioned *within* the assemblage, Barad (2007) speaks of “intra‐action” instead of “inter‐action.” An entity, such as a human individual, is not interacting with a structure, such as a legal system; rather, both are linked with each other (intra‐acting) because they are part of the same process. Therefore, the individual cannot cause the structure, nor can the structure cause the individual; both are a part of the very same process. A boundary between an entity and its environment is therefore always only a boundary between entities *within* the process, but not a boundary to entities outside the process.

### *Transversality: Becoming With*

During constant and immanent processes of intra‐action, the boundaries of the participating entities, such as humans or objects, are formed and maintained (Barad, 2003, 2007). Coole (2013) described that process as “ineluctably multiple and complex; variegated, folded, labyrinthine; and multi‐dimensional and multi‐scalar…. The point here is that entities, structures, objects all emerge as unstable, indeterminate assemblages that are composed of and folded into manifold smaller and larger assemblages” (p. 455). However, because there is no position outside the phenomenon, the separated entities are all transversally connected. The entities are perceived as *becoming with* (Braidotti, 2006; Haraway, 2008) one another instead of causing one another. Therefore, Haraway (1992, 2008) described the separated entities and networks of entities as *figurations*. Made in material‐discursive processes, the entities are not just material, discursive, or cultural. Therefore, humans are material‐discursive figurations, as are objects, structures, values, and meanings. A figuration can be perceived as fixed and real but not as ahistorical and merely natural. Further, from a new materialist perspective, a figuration cannot be investigated on its own. Research must take into account the intraconnected entities. Therefore, research on human relationships has to focus on humans *and* their situation in physical and discursive microenvironments *and* the material and discursive structures linked to that situation. Because all those entities are shaped in the same processes and within the boundaries of a specific entity, for example, a couple can be understood only if all intracontextual processes are included in the research. All processes and entities are therefore always posthuman.

### *Posthuman Agency*

New materialist theories question humanist notions of agency that are inherent in individuals. Instead, Barad (2007) has described agency as a part of the world's differentiation process. This process has the agency to form boundaries through intra‐action, which forms entities within a specific “space of possibilities” (Barad, 2007, p. 182). “Crucially, agency is a matter of intra‐acting; it is an enactment, not something that someone or something has” (Barad, 2007, p. 245). Similarly, Braidotti (2006) described agency as a part of constant forces and flows. This posthumanist understanding of agency again redefines the role of humans and structures built by humans in defining configurations like family.

I previously argued that entities are transversal since they are produced in the very same differentiation processes. Agency is, therefore, not to be found within a single entity, but within the intraconnected entities formed in the process. It has multiple locations within the relations of intra‐acting entities. From this perspective an individual does have the “space of possibility” to assign the meaning family to people or practices, but the boundaries of family are made together with the processes and entities they are becoming with, such as family formation practices, practices of maintaining family, or practices of dissolving family, which include humans, objects, buildings, values, discourses, media, and music. A collective process of agency (Coole, 2013) is configuring family within all these activities and entities.

These four basic qualities of new materialism (anti‐dualism, radical immanence, transversality, and posthuman agency) provide the foundation for a conception of contemporary configurations of family that are fixed and fluid at once. The following sections focus on the challenge of defining and researching those relationships configured as families and how they are repeatedly maintained and put into question.

## Consequences of New Materialism for Family Theory and Research

Applied to the field of family studies, several theoretical assumptions have consequences for the definition of family and for research on family processes: (a) Family relationships are not the product of context or the product of humans but are part of posthuman boundary making processes; (b) these processes are in constant transformation, which configures boundaries that are at once material and discursive, fixed and fluid; and (c) because families are not the product of humans, human structures, or discourses alone, the material and discursive contexts and environments of family processes become a part of the definition. From a new materialist perspective, then, family is a figuration; multifold processes and entities are part of and a consequence of everyday family processes that create the boundaries of the figuration “family.”

Thus, a theoretical definition of family from the perspective of new materialism can provide neither a fixed set of posthuman processes that form family nor a fixed set of entities that *are* family. Researchers can, however, formulate a complex theoretical definition of family boundaries, and they can provide concrete definitions via empirical material: For instance, the boundaries of family can be established through the structure of buildings (divided into rooms fit for a certain number of people), TV shows favoring a certain picture of family, positive or dismissive narratives about different family formations, means of transportation that allow commuting to (distant) family members, communication technologies, positive and negative experiences with the concept of family or communities that foster specific requirements regarding relationships between family members. All these structures, discourses, meanings, and personal experiences are processes that constitute the boundaries of specific relationships and therefore also the boundaries of families. However, these are not the cause of family or family boundaries; these are the transversally linked processes, “becoming with” the boundaries of family.

From the perspective of new materialisms family relationships are not made, constructed, or done by humans but are enacted by human and nonhuman entities that are part of material‐discursive differentiation processes. A focus on these numerous material‐discursive processes requires attention to the corporeality of family configuration and how family is formed as vitally material. Family members, structures, and environments appear in specific physical configurations. Family, therefore, is and has a body itself, which is separated into family members, humans, animals, or things by intra‐action. The goal is not simply to expand a notion of the (human) body to an assemblage like family, but to explicate configured boundaries as not just powerful concepts but also real in their materiality. For example, houses separating families may explain the material reality of family boundaries just as well as legal regulations that differ between institutionalized family forms and noninstitutionalized family forms. This perspective recognizes the material processes that forge families and family members.

### *Two Empirical Examples*

In this section I provide two brief empirical examples from my own research to illustrate the advantages of a new materialist approach for family studies in which (a) human family members are “becoming with” multiple other participants of the process; (b) because human family members are transversally linked to their environments, these environments are taken into account; (c) family is understood as a material and discursive formation; and (d) family is not a static entity but is in constant transformation as the boundaries of family are maintained, dissolved, or formed.

The first example is from a study of heterosexual couples during the transition to parenthood that examined the process of becoming a nuclear family. Using ethnography, I examined a process of activities and the human and nonhuman participants of those activities, for instance, the practices of trying to conceive a baby and the devices, knowledge, and behaviors they included. To name just a few, cycle charts or ovulation tests measure the most likely time of conception, and popular discourses on the Internet tell prospective parents about behavior or foods that aid conception. Being part of these practices of conceiving includes connection to numerous objects, knowledge, discourses, values, and other humans.

Making visible how much work conceiving, establishing evidence whether a pregnancy exists, announcing a pregnancy, maintaining a pregnancy, giving birth, and registering a child can be shows how, for example, the nuclear family is a figuration that has to be established and maintained in material and discursive processes (Schadler, 2013). Thus, in terms of new materialism, family is not only created by the humans involved; those humans and all the other included entities enact specific positions in the process of becoming a parent. In this research, both the work and the numerous entities included in the process become visible.

Another empirical example involves a group of three people in a polyamorous relationship. In this study, rather than investigate the detailed activities that happen within a short period of time, I focused on people and the processes they are becoming with over a longer period of time. In this case Mary, a member of this group, did not have a positive relationship with her biological parents and did not define them as her family. Nevertheless, when her father died, she inherited money, and the triad, which at the time was not yet living together, decided to use the money to buy a house and move in with one another. Searching for a house, they were constantly reminded that houses are built for two‐adult couples, as is furniture. For example, they had a bed custom made because furniture stores sell beds only for two people. During the practices of reading ads, surveying houses, and gaining information about property laws, they were configured as a family that does not fit predefined regulations. They nevertheless lived in an environment where polyamorous formations are allowed to live publicly, where poly‐communities existed, and where some help and counsel for “alternative” family configurations was available. They read about similar stories online, they discussed their problems with friends, and they found legal counsel.

Research from a new materialist perspective illustrates the entanglement of these processes over time and shows which other processes question and maintain the triad. It also illustrates how this formation is configured as “alternative” or “deviant” in posthuman everyday practices and how the triadic relationship is enabled, maintained, and normalized in activities within a poly‐community. Inequalities between different forms of family relationships become evident, as do environments that foster or mitigate differences between family configurations.

These two examples illustrate ethnographic research on relationships in which human relationships are embedded in material and discursive processes. The task of the new materialist researcher is to name the processes and the entities that configure specific human relationships. In both cases, I traced the world and the differentiation processes that the humans were embedded in and pictured the relationships and humans as inseparable from this world. Therefore, among others, the cycle charts, the houses, the furniture, discourses about couples, self‐definitions of family and legal regulations are a part of the relationship, because they are a part of the process that maintains the relationship between the humans.

This network of human and nonhuman entities is, however, temporary and ever‐changing. By tracking such a network over time, it becomes evident how some parts of the network remain rather stable, whereas other entanglements dissolve. Both empirical examples deal with transition periods (transition to parenthood and moving in together) and the relationships they include. Depending on the topic, either the description can focus on specific practices (e.g., trying to conceive a baby) and describe them in great detail, or it can focus on a broader illustration of humans and their contexts (e.g., polyamorous households). The outcome is situated processes that include specific activities and entities that form, maintain, or dissolve specific boundaries of family.

If we understand how complex the processes of becoming a nuclear family or a polyamorous network are, it becomes evident why the notion of family is a real figuration within the world; however, its boundaries also are constantly shifting and hard to grasp. Because there are so many activities and participants that contribute to forming, maintaining, and redefining these boundaries, reductionist accounts are able to measure only a part of this process, and they have a hard time keeping track of the shifting boundaries.

### *Consequences for Methodology and Empirical Research*

Consistent with the principles of new materialism, there is no set of rules for how to do new materialist research other than taking processes of becoming with into consideration. Every method that takes the transversal intraconnection of entities and processes into consideration and does not reduce the data to simple processes of causation can be suitable for research from a new materialist perspective. It is very important to new materialist researchers that the outcome of a study is not simply a representation of the research object (i.e., that which is studied) but a reference to both the research object and its entangled contextual processes. Thus, the research process itself also is a part of this processual context.

Although there are no strict rules for new materialist research, in recent years scholars have used several methodological strategies more frequently than others. One such strategy is to focus on methods developed in postmodern and poststructuralist research, such as autoethnography (Ellis, 2004; Ellis, Adams, & Bochner, 2010) and performance ethnography (Denzin, 2003a, 2003b). The tools used in this research are introspective writing, initiating performances, observing (self‐)performances and conversations. Another strategy is to focus on methods subsumed under the term *non‐representational research* (Vannini, 2015), which also includes collaborating with artists, activists, or social workers. These studies use diverse methods, such as ethnographies, artifact creation and analysis, and atmospheric ethnographies (for an excellent overview, see Vannini, 2015, reviewed elsewhere in this issue). Further, authors also have focused on methods developed in Deleuzian research (Coleman & Ringrose, 2013; Hendricks & Koro‐Ljungberg, 2015; Taguchi, 2012). These authors combine interviews, ethnographies, or action research with Deleuzian theory and ontology. In these three strategies, it is also important to transgress traditional forms of academic writing. A fourth strategy is to make use of the concept of exteriority within (Barad, 2007) by using rather analytical research methods without creating a representationalist argument (Schadler, forthcoming). Research methods become apparatuses (Barad, 2007), which create specific diffractions and boundaries. The task is to include the research environment in the analysis of the research object. From this perspective, the research environment (e.g., the research institution, the department philosophy, the career stage of the researcher, the funding of the research) is an important intracontext. Therefore, these contexts may influence the research apparatus used. For instance, PhD students may be more restricted by specific conventions of a research department than tenured professors are. The funding of the research is important for the amount of data that can be collected or analyzed. Therefore, there cannot be a set of rules regarding how new materialist ethnographies must be conducted. This fourth research strategy also allows for rather traditional forms of writing.

All four strategies include an analysis that is sensitive to (shifting) boundaries, diffractions created in the research process and posthuman processes, which I consider the most important step in new materialist analysis. For new materialist research, how to think with the data and how to present a research outcome are most important.

In my own work I consider ethnographies that include multiple methods of inquiry (e.g., interviews, observations, web analysis) to be a suitable way to research family processes and boundaries. In a process I call referencing, I analyzed manifest and latent intracontextual or posthuman processes and intraconnected or transversal entities (Schadler, forthcoming). To disseminate my research in articles or presentations, I rebuild worlds in written words or visual data. These presentations are not considered a representation of the research object; rather, they are the research outcome and its intracontextual processes in its current form of becoming.

### *Contributions of New Materialist Research*

The strengths of research from a new materialist perspective are that it encounters relationships as embedded in numerous posthuman processes and it does not predominantly investigate specific processes, such as (intersecting) structures, individual meanings, discourses, or values. Although research that focuses on specific processes is important, the new materialist perspective adds a more open perspective by providing a nonreductionist approach with which to investigate the complex entanglement of numerous processes at once.

The claim to focus on entangled processes and nonreductionist research is no novelty to family studies. In particular, symbolic interactionist research investigates complex situated processes (LaRossa & Reitzes, 2004). However, in interactionist research the production of meaning and agency are centered in the individual, who has a preexisting disposition to process knowledge and meaning. The boundaries of family, therefore, always are negotiated by predefined human individuals, even when individuals negotiate complex structures. New materialist research does not define the human as the center of family boundaries, but the human and many other entities are differentiated and enacted in the posthuman processes of making family boundaries. The agency needed to create the boundaries of family is in the process and in every involved entity. As such, the concept of transversality includes a multilocal concept for the negotiation of meaning, knowledge, and agency.

Similar to life course theories (Elder, 2006), new materialisms take the links between socioeconomic structures and individual lives into account. However, because of its stronger focus on microprocesses, new materialism expands this perspective by adding situational contexts to the investigation process. During new materialist research, structures and biographies become components of a situation, next to specific local physical and social environments. The approach is able to show how all these social and material posthuman intracontextual processes become visible in a specific situation (e.g., in the process of searching for a house), as shown in the previously described example. Family relationships not only are expected to be linked with (analytically separate) contexts but also are perceived as an inseparable part of a specific constellation of intracontextual processes.

Similar to family systems theory, new materialisms perceive family members, individuals, or bodies of individuals as creating a context of family relationships along with structural contexts but consider these processes as less mechanistic and deterministic. New materialisms define boundaries as stable enough to allow iterations of specific relationships in situated contexts, but also as fluid enough to undergo constant transformations.

Feminist, poststructuralist, postmodern, and queer theories also share anti‐dualism and radical immanence foundations with new materialisms, although some new materialist researchers critique those theories for focusing too much on language and not focusing enough on material processes (Barad, 2007). In fact, the term *new materialism* came into use as a critique of these anti‐dualist theories and as an attempt to refocus the research on social, discursive, and material processes as inseparable activities (Dolphijn & Van der Tuin, 2013).

A general advantage of new materialist research is that it is able to look at processes that are invisible to survey research by taking both important beings and objects into consideration. Every process and entity that maintains, configures, or questions family relationships can be incorporated into the definition of family. For instance, the influence of technology or animals on relationships, or even human relationships to animals or technological devices, provides a platform for research on relationships that are seldom considered in family research.

However, while every new materialist analysis includes both physical environments and nonhuman components, not every study that includes nonhuman actors is a new materialist study. Symbolic interactionist research can include family members who assign the meaning “family” to an animal or a thing, and these entities can even become very important parts of family processes. Interpretative research can also focus on technology, such as mobile phones, and how they mediate family relationships. In new materialist research, however, all entities that maintain the boundaries of family are part of the family, and therefore it is obligatory to research posthuman processes, because within intraconnected contexts, humans and other entities are inseparable from each other.

## Conclusion

In this article, I have proposed adding new materialisms (e.g., Barad, 2007; Braidotti, 2002; Coole, 2013; Haraway, 2008) to current theoretical approaches in family science. After reviewing the theory of new materialism and its four basic concepts (anti‐dualism, radical immanence, transversality, and posthuman agency), I defined family as a figuration and hence the sum of situated activities and the entities that participate in those activities. The material and discursive boundaries of family are created within transversal and posthuman processes. Family is therefore not defined by its human members and by the inherent abilities these human members have (e.g., the ability to reason or to assign meaning). Human families include family members, all the activities they are a part of, and all the entities that are transversally linked to the family members. As shown in the two empirical examples previously discussed, family can be configured by activities that include devices, knowledge, texts, other humans, political discourses, legal texts, values, or previous experiences. All those activities and material‐discursive or naturecultural entities are becoming together and form, maintain, or dissolve the boundaries of family.

By taking these multiple entanglements into consideration, new materialist research could further advance the understanding of family relationships and how they become durable. New materialisms are nonreductionist research approaches that take into account the complex relations of everyday life, such as the connection of emergent technologies and family structures; however, many of the processes that become visible from a new materialist perspective have not gained enough attention, such as research on material environments—for example, architecture and furniture—and how they intra‐act with family life.

As family studies scholars seek to understand contemporary family configurations, we need to understand the complex intra‐action of numerous activities and entities that constantly rework diverse definitions of families while family itself still remains an important institution. I have suggested that current configurations of family living are constantly broadening, although traditional forms of family also endure. Research from the perspective of new materialism can provide insight into these already well‐investigated family forms. Even nuclear families may appear in a new light when their embeddedness and intra‐action with other entities and processes are examined. In making posthuman processes visible, new materialist approaches make evident that multiple family configurations exist and that families are not only a set of humans and their relationship to each other but also the activities and entities that configure, maintain, and question those relationships. The new materialist idea of family as a figuration, not a form, has the potential to stimulate innovations in family theory, research, and practice.

## Author Note

The research was funded by an Erwin‐Schrödinger‐Scholarship of the Austrian Science Fund (Grant No. J3451‐G22). The scholarship is cofunded by the Marie‐Curie‐Actions of the European Commission. I thank Björn Ganslandt, Thomas Herscht, and Ruth Mueller for comments on previous versions of the manuscript. I thank Mike Holohan for language support.
